# Hospital Progress in Paris.—I

**Published:** 1905-05-06

**Authors:** 


### HOSPITAL PROGRESS IN PARIS.—I.

By the Author of "Hospital Expenditure: The Commissariat."

In view of the projected visit of English medical men to Paris special interest attaches at the present moment to the system of hospital administration which will there be brought under their notice. It is well known to the student of hospital systems that in Paris a central bureau under the name of "L'Assistance Publique," is charged with the entire responsibility of relieving the sick and aged poor. An admirable account of the machinery of the "Assistance Publique" will be found in "Burdett's Hospitals and Asylums of the World," by aid of which a clear impression can be gained of this unique system as a preparatory step to personal inspection of the service. The "Assistance Publique" combines the functions divided over here between the hospital boards and the poor-law officials, and since some people in the present day are found to advocate the adoption of a similar fusion in London, it is instructive to note the advantages and drawbacks which over 50 years of experience have shown it to possess.

The provisions of the law of 1849, which constituted the administration of the Assistance Publique, have been expanded by subsequent decrees as occasion has arisen, but the hospital service, established as it was at a period preceding the great medical and surgical triumphs of the nineteenth century, has of late years proved more and more inadequate, and in 1902 the first steps towards a complete reorganisation were definitely taken. It is through the medium of this reorganisation that some perception may be gained of the strength and weakness of the centralising system, in practical working order.

Although no new hospitals have been built in Paris since the opening of "Tenon" in 1878, the increase in the number of beds is greater in proportion than the increase in the urban population. In 1882 Paris had one bed to every 216 inhabitants; in 1902 there is one bed to every 185 inhabitants, the number of beds having increased from 10,375 to 14,659 during this period.* The growth of the suburban region round Paris, the continual increase in the number of patients sent up to the metropolis from country districts (though this is discountenanced by law), and, more than all, the increasing disposition of the lower bourgeois class to resort to hospital aid, have combined to send up the hospital *clientèle* in a ratio out of all proportion to the increase in population. The figures are of great interest as showing that this tendency to make unscrupulous use of hospital accommodation designed for the needy is not confined to our own metropolis and has no necessary connection with the voluntary hospital system. In the course of ten years the population shows an increase of about 500,000. During the same period the number of hospital "days of treatment" has risen to the extent of 1,600,000.

The immediate result of this influx of patients has been the necessity for litter beds or *lits supplémentaires*, the number of which varies according to circumstances. In 1902 the maximum was reached in February, when 1,866 *brancards* were occupied in the hospitals throughout Paris, the minimum of 501 occurring in the month of July. The disadvantages of combining the functions of medical relief and municipal charity are forcibly illustrated by this system which seems inevitable to Poor-law relief.† The claimant for municipal charity may not be rejected and, in consequence, the surgical and medical work of the wards may be seriously impeded. On the occasion of a recent visit on a mild spring day to the Hôtel Dieu the men's surgical ward of 26 beds contained 28 patients—two in litter beds; the air was heavy, and, to make matters worse, out of 14 long windows in the crowded ward, 13 were tightly closed.

In dealing with the situation exposed by this deficiency of accommodation the strong points of a central system become at once manifest. The needs of the city can be considered as a whole, overlapping can be avoided, weak points can be strengthened, and every link in the great chain of medical relief can be tested and made good. Hospital work is raised from the position of rightly or wrongly directed private effort into the plane of statesmanship. *Theoretically* there can be little question as to the advantage of a central expert authority in matters of such deep importance to the State, and London, with its strangely unequal distribution of hospitals, illustrates more forcibly perhaps than any other city the natural result of a disintegrated system.

The revision of the Parisian hospital service owes its initiation to M. Mourier, and, on his death in 1902, after exhaustive labours in the interest of reform, it has been carried on with zeal and energy by M. Mesureur, the present able Director-General of the "Assistance Publique."

* In London the proportion in 1902 was one bed to 470 inhabitants, but this is exclusive of the 15,120 beds in Poor-law infirmaries, nearly all of which are occupied by cases such as are relieved in Paris hospitals.

† In 1902 the number of certified beds at St. Mary's, Islington, Infirmary was only 473; and the daily average of in-patients was far in excess. At the Wandsworth and Clapham Infirmary there were 618 beds and an average of 663 in-patients.

The main difficulty in the way of instituting reforms long perceived to be urgent was, of course, financial. The sums at the disposal of the Central Administration in aid of the "extraordinary expenses" of the hospital service were appropriated in advance by the ever-increasing cost of keeping the old hospitals in repair and by improvements imperatively demanded in the advance of science. Successive commissions sitting over a period of six years reported one after another the need for "extraordinary disbursements," advancing always in amount, till from the 60,000,000 frs. considered excessive in 1897, a total of 84,000,000 frs. (£3,360,000) was found to be but barely sufficient to carry out the reconstructions and additions urgently required in 1903. Some of the Parisian hospitals had, in fact, reached a stage of dilapidation in which they had become a positive source of danger to the community. La Pitié, Broussais, Andral, Bichat, Cochin-Ricord, Aubervilliers were one and all unfit for the reception of patients, while additional hospitals were indispensable to the public requirements. The "Assistance Publique" had, in fact, reached that period of crisis in hospital construction which has faced within the last few years the voluntary hospitals in London, and which still confronts several of our leading institutions.

To raise money by the imposition of a new tax was the obvious method, a method rejected almost nnanimously by the municipality of Paris. It has remained for M. Mesureur to place the necessary reforms within the range of practical politics, and this he has done in two ways. He has first divided the operations, and by grappling for the moment only with the most urgent reforms reduced the capital required to 45,000,000 francs (£1,800,000). Secondly, by a clever financial scheme, he procured a loan, repayable in eight years and secured on the property of the "Assistance Publique." The projected works are now in active progress. They comprise:—New hospital in the grounds of La Salpêtrière to replace La Pitié, 760 beds; hospital for contagious diseases to replace Aubervilliers, 480 beds; new hospital on right bank of Seine, 800 beds; enlargement of hospital at Berch-sur-Mer, 300 beds; reconstruction of the hospital Cochin-Ricord, 1,000 beds; enlargement of sanatorium at Hendaye, 200 beds; reconstruction of the Hôpital Broca, 304 beds. The effect of this first series of operations will be to add 1,547 beds to the existing hospital accommodation. Five series of important repairs and re-equipment works will be carried out at the same time.

We have said that theoretically the central administration has overwhelming advantages in dealing with hospital construction. The long delays, however, in setting the necessary machinery to work, and the want of foresight which has permitted so many institutions to fall simultaneously into decay, illustrate the reverse side of the question. And this is shown even more forcibly in the second great reform, that of the "hospital *personnel*," which is at length being gradually accomplished.

(*To be continued.*)

